# Supplementary material for: Effects of supervised high-intensity hardstyle kettlebell training on grip strength and health-related physical fitness in insufficiently active older adults: the BELL pragmatic controlled trial
Source: BMC Geriatr. 2022 Apr 22;22:354. doi: 10.1186/s12877-022-02958-z (PMC9026020; doi:10.1186/s12877-022-02958-z)
Supplement: Supplementary file 2 — Additional file 2. [file 12877_2022_2958_MOESM2_ESM.pdf]

The BELL (Ballistic Exercise of the Lower Limb) trial: A repeated measures, single cohort, pragmatic hardstyle kettlebell training program to improve grip strength, health-related physical fitness, and quality of life in sedentary older adults.

Australian New Zealand Clinical Trials Registry (ID: ACTRN12619001177145).

CONSERVE-CONSORT Extension

Item	Item Title	Description	Page No.
I.	Extenuating circumstances	Unavoidable modifications in response to the emergence of the COVID-19 pandemic and associated local government restrictions after commencement of the intervention.	4, 7-8
II.	Important modifications	a. Supervised group-based training was replaced with home-based training from week 7 of a 12-week intervention.	7-8
		b. From week 7, participants were limited to using only three kettlebells (of 2-3 different masses), with access to heavier kettlebells (up to 80 kg) unavailable. Only two participants had a kettlebell mass >32kg.	8
		c. From week 7, training videos were provided by YouTube, or instruction was given via email or Facebook (private group page) when video was not required.	8
		d. Test 4, scheduled to take place in Week 22 (at the end of week 8 of the intervention), took place in week 19 (6 weeks of intervention). The testing scheduled was altered, with some outcomes e.g., DXA, not permitted due to close contact restrictions.	5, 8
		e. Test 5 was delayed from week 26 to week 29. Participants 70+ years of age were only permitted to complete three outcome measures, under altered conditions (outdoors). Participants <70 years were permitted to complete a limited number of outcomes.	5, 8
III.	Responsible parties	The lead author (NM) planned modifications, which were reviewed by WH/JK, and approved by the Chair of the Bond University Human Research Ethics Committee (BUHREC).	n/a
IV.	Interim data	n/a	

CONSORT Number and Item		No change	Impact*	Mitigating strategy**	Page No.
1	Title and abstract	✓			
2	Introduction	✓			
3	Methods: Trial Design	✓			

4	Methods: Participants	✓			
5	Methods: Interventions		✓	3x face-to-face group sessions + 2x home-based training sessions each week, became 5x home-based sessions weekly, with training videos provided via YouTube. Participants were given two additional kettlebells (three in total) for the remainder of the trial. Collection of training data changed from paper-based to online (via Survey Monkey). The instructor maintained daily communication via email and private Facebook group.	8-9
6	Methods: Outcomes		✓	Test 4 occurred two weeks earlier than planned. Due to COVID-19 close contact restrictions, only the following outcome physical measures were permitted to be collected in week 19 (Test 4): i) grip strength, ii) stair climb, iii) vertical jump, and iv) 5x floor transfer.<br>Test 5 was delayed by 3 weeks. Ethical clearance, for participants 70+ years of age, was granted for only the three measures during Test 5 (week 29), which were conducted outdoors: i) grip strength, ii) 6-min walk, and iii) 5x floor transfer. Questionnaires were completed online using Survey Monkey. For participants <70 years of age, Test 5 was conducted under the same conditions as Tests 1-3 (following COVID-19 guidelines regarding use of PPE, hand-washing and social distancing).	5, 8
7	Methods: Sample Size	✓			
8-10	Methods: Randomisation	✓			
11	Methods: Blinding	✓			
12	Methods: Statistical methods	✓			
13	Results: Participant flow	✓			
14	Results: Recruitment	✓			
15	Results: Baseline data	✓			
16	Results: Numbers analysed	✓			
17	Results: Outcomes and estimation	✓			

18	Results: Ancillary analyses	✓			
19	Results: Harms	✓			
20	Discussion: Limitations				
21	Discussion: Generalisability	✓			
22	Other information: Registration	✓			
23	Other information: Protocol	✓			
24	Other information: Funding	✓			

*Aspects of the trial that are directly affected or changed by the extenuating circumstance and are not under the control of investigators, sponsor or funder.

**Aspects of the trial that are modified by the study investigators, sponsor, or funder to respond to the extenuating circumstance or manage the direct impacts on the trial.

Cite as: *Orkin, A. M., Gill, P. J., Ghersi, D., Campbell, L., Sugarman, J., Emsley, R., ... & CONSERVE Group. (2021). Guidelines for Reporting Trial Protocols and Completed Trials Modified Due to the COVID-19 Pandemic and Other Extenuating Circumstances: The CONSERVE 2021 Statement. JAMA.*
