# Supplementary material for: Effects of supervised high-intensity hardstyle kettlebell training on grip strength and health-related physical fitness in insufficiently active older adults: the BELL pragmatic controlled trial
Source: BMC Geriatr. 2022 Apr 22;22:354. doi: 10.1186/s12877-022-02958-z (PMC9026020; doi:10.1186/s12877-022-02958-z)
Supplement: Supplementary file 3 — Additional file 3. [file 12877_2022_2958_MOESM3_ESM.pdf]

The BELL (Ballistic Exercise of the Lower Limb) trial: A repeated measures, single cohort, pragmatic hardstyle kettlebell training program to improve grip strength, health-related physical fitness, and quality of life in sedentary older adults.

Australian New Zealand Clinical Trials Registry (ID: ACTRN12619001177145).

CONSORT Checklist of items for reporting pragmatic trials

Section	Item	Standard CONSORT description	Extension for pragmatic trials	Addressed on page number/ heading
Title and abstract	1	How participants were allocated to interventions (e.g., "random allocation," "randomised," or "randomly assigned")		N/A
**Introduction**				
Background	2	Scientific background and explanation of rationale	Describe the health or health service problem that the intervention is intended to address and other interventions that may commonly be aimed at this problem	p2-4, 1. Introduction
**Methods**				
Participants	3	Eligibility criteria for participants; settings and locations where the data were collected	Eligibility criteria should be explicitly framed to show the degree to which they include typical participants and/or, where applicable, typical providers (e.g., nurses), institutions (e.g., hospitals), communities (or localities e.g., towns) and settings of care (e.g., different healthcare financing systems)	p5, 2.3. Participants p32, 5. Strengths and limitations
Interventions	4	Precise details of the interventions intended for each group and how and when they were actually administered	Describe extra resources added to (or resources removed from) usual settings in order to implement intervention. Indicate if efforts were made to standardise the intervention or if the intervention and its delivery were allowed to vary between participants, practitioners, or study sites<br><br>Describe the comparator in similar detail to the intervention	p7-8, 2.4. Exercise intervention, p8, 2.5. Control activities Supplementary data
Objectives	5	Specific objectives and hypotheses		p2-3 1. Introduction
Outcomes	6	Clearly defined primary and secondary outcome measures and, when applicable, any methods used to enhance the quality of measurements (e.g., multiple observations, training of assessors)	Explain why the chosen outcomes and, when relevant, the length of follow-up are considered important to those who will use the results of the trial	p4, 2.1. Study design and sample size

Section	Item	Standard CONSORT description	Extension for pragmatic trials	Addressed on page number/ heading
Sample size	7	How sample size was determined; explanation of any interim analyses and stopping rules when applicable	If calculated using the smallest difference considered important by the target decision maker audience (the minimally important difference) then report where this difference was obtained	p4, 2.1. Study design and sample size
Randomisation—sequence generation	8	Method used to generate the random allocation sequence, including details of any restriction (e.g., blocking, stratification)		N/A
Randomisation—allocation concealment	9	Method used to implement the random allocation sequence (e.g., numbered containers or central telephone), clarifying whether the sequence was concealed until interventions were assigned		N/A
Randomisation—implementation	10	Who generated the allocation sequence, who enrolled participants, and who assigned participants to their groups		N/A
Blinding (masking)	11	Whether participants, those administering the interventions, and those assessing the outcomes were blinded to group assignment	If blinding was not done, or was not possible, explain why	p4, 2.1. Study design and sample size
Statistical methods	12	Statistical methods used to compare groups for primary outcomes; methods for additional analyses, such as subgroup analyses and adjusted analyses		p15, 2.7. Statistical analysis
**Results**				
Participant flow	13	Flow of participants through each stage (a diagram is strongly recommended)—specifically, for each group, report the numbers of participants randomly assigned, receiving intended treatment, completing the study protocol, and analysed for the primary outcome; describe deviations from planned study protocol, together with reasons	The number of participants or units approached to take part in the trial, the number which were eligible, and reasons for non-participation should be reported	p16, Fig. 4 Participant flow
Recruitment	14	Dates defining the periods of recruitment and follow-up		p5, Fig .1 Study timeline
Baseline data	15	Baseline demographic and clinical characteristics of each group		p15-16, 3.1 Participant characteristics at baseline
Numbers analysed	16	Number of participants (denominator) in each group included in each analysis and whether analysis was by “intention-to-treat”; state the results in absolute numbers when feasible (eg, 10/20, not 50%)		p15, 2.7. Statistical analysis p16, 3. Results

Section	Item	Standard CONSORT description	Extension for pragmatic trials	Addressed on page number/ heading
Outcomes and estimation	17	For each primary and secondary outcome, a summary of results for each group and the estimated effect size and its precision (e.g., 95% CI)		p18-19, Table 2<br>p20, Table 3
Ancillary analyses	18	Address multiplicity by reporting any other analyses performed, including subgroup analyses and adjusted analyses, indicating which are prespecified and which are exploratory		N/A
Adverse events	19	All important adverse events or side effects in each intervention group		p23, 3.3.8.<br>p32, 4.9.<br>Adverse events
**Discussion**				
Interpretation	20	Interpretation of the results, taking into account study hypotheses, sources of potential bias or imprecision, and the dangers associated with multiplicity of analyses and outcomes		p24-30, 4. Discussion<br>p30, 5. Strengths and limitations
Generalisability	21	Generalisability (external validity) of the trial findings	Describe key aspects of the setting which determined the trial results. Discuss possible differences in other settings where clinical traditions, health service organisation, staffing, or resources may vary from those of the trial	P30, 5. Strengths and limitations
Overall evidence	22	General interpretation of the results in the context of current evidence		p24-30, 4. Discussion

Cite as: *Zwarenstein M, Treweek S, Gagnier JJ, Altman DG, Tunis S, Haynes B, Oxman AD, Moher D for the CONSORT and Pragmatic Trials in Healthcare (Practihc) group. Improving the reporting of pragmatic trials: an extension of the CONSORT statement. BMJ 2008; 337;a2390.*
